# Health literacy and frailty: the mediating role of instrumental activities of daily living

**DOI:** 10.1111/psyg.70010

**Published:** 2025-02-10

**Authors:** Keisuke Nakamura, Tomohiro Sasaki, Yoshiharu Yokokawa, Shinobu Yokouchi

**Affiliations:** ^1^ Department of Physical Therapy School of Health Sciences, Shinshu University Matsumoto Japan; ^2^ Health Promotion Division Matsumoto City Hall Matsumoto Japan

**Keywords:** activities of daily living, frailty, health literacy, interventions, older adults

## Abstract

**Introduction:**

Japan has the fastest ageing population worldwide, with a high prevalence of frailty. This study aimed to investigate the impact of communicative and critical health literacy (CCHL) on the progression of frailty in older adults participating in community‐based programs over 1 year, and whether instrumental activities of daily living (IADL) mediate this relationship.

**Methods:**

This retrospective cohort study used data from the Matsumoto City Frailty Prevention Project, involving 373 older adults aged 65 years and over. Health literacy was measured using the CCHL scale, and IADL was assessed using the Tokyo Metropolitan Institute of Gerontology Index of Competence (TMIG‐IC), which includes five items of IADLs. Frailty was classified using the Japanese version of the Cardiovascular Health Study (J‐CHS) criteria. Mediation analysis was used to evaluate the role of IADL in the relationship between health literacy and frailty.

**Results:**

Participants with higher CCHL had significantly better IADL scores (coefficient = 0.127, *P* = 0.043) and were less likely to progress to frailty (odds ratio: 0.546, *P* = 0.009). Mediation analysis revealed that IADL accounted for approximately 10.7% of the total effect of health literacy on frailty progression (*P* = 0.030).

**Conclusion:**

Higher CCHL reduces the risk of frailty progression, with IADL playing a mediating role. Interventions targeting both health literacy and IADL may effectively prevent frailty in older adults.

## INTRODUCTION

Populations across the world are ageing at an increasing rate, especially in Japan, which has the fastest ageing society in the world and high prevalence of frailty.^1^, ^2^ Thus, frailty is a significant social and medical issue, characterised by age‐related decline in physiological reserves, leading to adverse outcomes, including disability and death, often triggered by mild stress.^3^, ^4^ Early diagnosis and treatment may reverse frailty progression, highlighting the need for timely identification and intervention.^5^

As per the World Health Organization ‘Health literacy represents the cognitive and social skills which determine the motivation and ability of individuals to gain access to, understand and use information in ways which promote and maintain good health.’^6^ Thus, health literacy has three levels: (1) functional—basic reading and writing skills; (2) communicative—applying new information to changing circumstances; and (3) critical—analyzing and using information to manage different circumstances.^7^

Low health literacy leads to increased healthcare utilisation and costs,^8^ while higher health literacy enables better information gathering and healthier behaviours.^9^ In older adults, higher health literacy is associated with fewer chronic conditions and better physical function;^9^ thus, health literacy may play a critical role in health management and quality of life maintenance.

Despite these established associations, the direct relationship between health literacy and frailty remains understudied. A previous study demonstrated that higher health literacy was associated with a lower risk of frailty progression over 4 years.^10^ However, this study only used the Kihon Checklist as a frailty assessment, without incorporating physical frailty assessments such as the well‐validated Japanese version of the Cardiovascular Health Study (J‐CHS) criteria, which is considered the gold standard for frailty evaluation.^10^ Another study reported that limited health literacy increased the risk of pre‐frailty by 1.4 times over a period of 2 years; however, the study focused only on functional literacy.^11^

The role of health literacy in the management of older adults' health has been increasingly recognised. However, the mechanisms involved in the effect of health literacy on frailty progression are still not fully understood. Specifically, the role of instrumental activities of daily living (IADL) in the effect of health literacy on frailty, remains underexplored. IADL refers to a broader range of daily activities that reflect physical and social independence and has been reported to be associated with both health literacy and frailty.^12^, ^13^, ^14^ IADL decline increases frailty risk and impairs physical function.^15^ Higher health literacy may contribute to the maintenance and improvement of ADLs, thus, delaying frailty progression. However, the role of IADL as an associated factor or mediator in the relationship between health literacy and frailty, remains unclear.

Understanding the role of IADL in this relationship is critical for the development of better frailty prevention and management strategies. Furthermore, focusing on communicative and critical health literacy (CCHL), rather than only functional literacy, may provide deeper insights into improving health management and preventing frailty.

We hypothesised that: (1) older adults with lower CCHL literacy would show poorer frailty outcomes after 1 year, independent of potential confounders; and (2) IADL would serve as a mediating factor in the relationship between health literacy and frailty progression. Therefore, the aim of this study was to investigate the impact of CCHL on the progression of pre‐frailty and frailty in older adults over 1 year. We also characterised the role of IADL in this relationship.

## METHODS

### Study design and participants

In this retrospective cohort study, data from surveys conducted for the Matsumoto City Frailty Prevention Project in 2021 and 2022 were used to assess the factors contributing to frailty progression. The program included a single frailty assessment and a one‐time frailty prevention lecture. The lecture provided information on key aspects of frailty prevention, including exercise, nutrition, medication management, and oral health. All participants attended both the assessment and the lecture, ensuring full participation in the program. The survey was focused on older adults residing in Matsumoto City, Nagano Prefecture, Japan.

**Figure 1:**
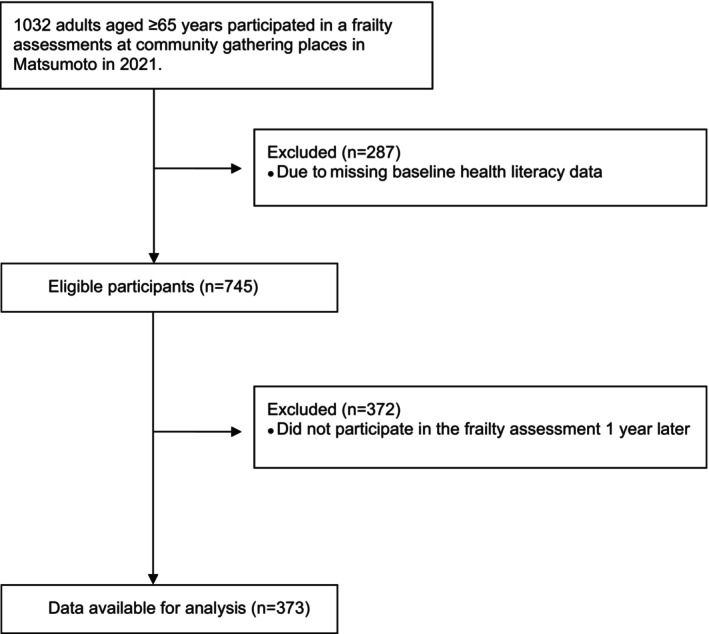
Patients' recruitment and flow diagram.

We included individuals residing in Matsumoto City aged ≥65 years who participated in frailty and health literacy assessments at community gathering places in Matsumoto City in 2021. The participants attended a 1‐day nutritional and exercise guidance session or a frailty‐related medication lecture. The inclusion criteria were: (1) aged ≥65 years; and (2) participation in both frailty and health literacy assessments at baseline. The exclusion criterion was non‐participation in the frailty assessment at the 1‐year follow‐up. The participant recruitment flowchart is presented in Fig. 1. The study was approved by the ethics committee of Matsumoto City Hospital (protocol number, 03–5; approval date, 22/06/2021), and conducted as per the guidelines of the Declaration of Helsinki. In this observational study, the data were anonymised and obtained from non‐invasive assessments conducted at community gathering places; therefore, the need for individual participant consent was waived. However, the participants were informed about the study and provided relevant information. Furthermore, the participants were informed that they could withdraw from the study at any time.

### Measurement

#### Baseline assessment

Health literacy was measured using the CCHL scale.^16^ This scale measures three aspects of health literacy, including ability to access, understand, and use health information. The participants were enquired about their ability to: (1) obtain health‐related information from various sources; (2) extract the required information; (3) understand and communicate the information obtained; (4) assess the reliability of the information; and (5) make decisions based on the information, specifically in the context of health‐related issues. Each item was rated on a five‐point Likert scale: 1 (strongly disagree); 2 (somewhat disagree); 3 (undecided); 4 (somewhat agree); and 5 (strongly agree). The scores of the five items were then averaged. Participants were classified into two groups based on their average score into high health literacy (high HL; with an average score of ≥4) and low health literacy (low HL; with an average score of <4) groups. This classification was based on a previous study that reported that the median health literacy score of the population was 4.^16^ Using this threshold, we aligned our classification with established research practices.

Other measures assessed in the study were basic demographics (age, sex, and years of education) and the number of chronic diseases (heart disease, cerebrovascular disease, respiratory disease, diabetes, cancer, rheumatoid arthritis, and osteoarthritis). The years of education were categorised into four groups (Group 1–reference, approximately 9 years; Group 2, approximately 12 years; Group 3, approximately 15 years; and Group 4, others). Chronic diseases were defined as “yes” if diagnosed by a physician. Subjective cognitive function was assessed using three questions from the Kihon Checklist: “Do people around you say you keep repeating the same questions or make similar comments about your forgetfulness?”, “Can you look up phone numbers to make phone calls by yourself?”, and “Do you find yourself not knowing today's date?”^17^ Subjective cognitive decline was defined as at least one negative response to these questions.^17^

Social factors, including living arrangements (living alone or not) and employment status (currently employed or not), were also assessed. Additionally, at baseline, participants were assessed using the Tokyo Metropolitan Institute of Gerontology Index of Competence (TMIG‐IC) (five instrumental ADLs items) for IADL.^18^ If the answer was “yes,” one point was added; if the answer was “no,” zero points were added. The total score ranged 0–5, with higher scores indicating greater functional abilities.

Normal gait speed and grip strength were assessed as indicators of physical function. Gait speed was calculated based on the time(s) required to walk a distance of 5 m. The participants were instructed to walk at a comfortable pace in an unobstructed hallway. Patients were permitted to use assistive devices including walkers and canes. Grip strength was measured on both sides of the body and calculated as the average of the left‐ and right‐hand grip strength measurements. Participants were also assessed for fatigue, physical activity, and weight loss of more than 2–3 kg within the last 6 months. The revised J‐CHS criteria were used to assess frailty.^19^ According to the revised J‐CHS criteria, participants were classified into three frailty status groups: frail (≥3 points), pre‐frail (1–2 points), and robust (0 points). The criteria included the following components: (i) shrinking, defined as unintentional weight loss of ≥2 kg in the past 6 months; (ii) weakness, defined as grip strength <28 kg for men and < 18 kg for women; (iii) exhaustion, defined as feeling tired without reason in the past 2 weeks; (iv) slowness, defined as gait speed <1.0 m/s; and (v) low activity, defined as not engaging in moderate or low levels of physical exercise to improve health.^19^ For this analysis, participants classified as pre‐frail or frail were combined into a single group, while, those classified as robust were analyzed separately, resulting in two comparison groups based on frailty status.

#### Follow‐up assessment

One year after the baseline assessment, the presence or absence of pre‐frailty and frailty was assessed again in community gathering places using the J‐CHS criteria. Medical and long‐term care costs from baseline to 1 year were identified using the health insurance claims data and long‐term care insurance claims data. The Kokuho Database (KDB) contains health insurance claims data (e.g., monthly claims for patients' diagnoses, procedures, and medications) and long‐term care insurance claims data.

### Statistical analyses

We presented the sociodemographic characteristics and outcome measures of the high HL and low HL groups as descriptive statistics. For categorical variables, including sex, employment status, and living arrangements, we reported the number and percentage of participants. For continuous variables, including age and IADL, we provided means (standard deviation), and median (25th and 75th percentiles).

We used regression analyses to assess the effects of health literacy on IADL and frailty. Linear regression was used to assess the relationship between health literacy and IADL, adjusting for confounders including age, comorbidities, education level, cognitive function, living arrangements, employment status, and baseline frailty. Results of these analyses are presented as estimates, standard errors, and *P*‐values.

We then applied logistic regression to examine the association between health literacy and frailty status after 1 year, using the same set of covariates. Results are reported as odds ratios (OR) with 95% confidence intervals (CI).

Mediation analysis was conducted to assess how IADL affected frailty status changes. We decomposed the total effect of health literacy on frailty into the average direct effect (ADE) and the average causal mediation effect (ACME), with 95% CIs obtained through 1000 bootstrap replications. A mediation effect of IADL was deemed present if the bootstrap 95% CI did not include zero and the regression coefficients were statistically significant. The mediated proportion was calculated as the ratio of the ACME to the total effect.

All statistical analyses were performed using R software (version 4.3.2; R Core Team, 2023). Missing values were imputed using the missForest algorithm,^20^ implemented via the “missForest” package. Mediation analyses were performed using the “mediation” package,^21^ and the threshold for statistical significance was set at *P* < 0.05, unless otherwise specified.

## RESULTS

Overall, 373 older adults were included in the study and underwent frailty assessments over 2 years. Of these, 15.6% were male, 56.3% were classified as robust, and 43.7% were pre‐frail or frail at baseline (Table 1). Participant characteristics were similar between the high and low HL groups, except for age and employment status. Those in the low HL group tended to be older (79.7 ± 5.8 vs. 76.9 ± 6.4 years) and have a lower employment rate (6.8% vs. 16.1%) compared with those in the high HL group (Table 1). The baseline comparison between the two groups (*n* = 372 and *n* = 373, respectively) revealed no statistically significant differences in participant characteristics between participants who could not be followed up for a frailty assessment after 1 year and those who could be followed up (Table S1). Participants who could not be followed up were excluded from the final analysis.

**Table 1:** Patients' demographics at baseline

	High HL group	Low HL group	NMV
	*N* = 211	*N* = 162	
Age			1
Mean ± SD	76.9 (6.4)	79.7 (5.8)	
Median (25th and 75th percentiles)	77.0 (72, 82)	80.0 (76, 84)	
Sex			0
Male	15.6% (33)	17.3% (28)	
Female	84.4% (178)	82.7% (134)	
Educational background			3
9 years (Group 1)	13.9% (29)	19.3% (31)	
12 years (Group 2)	58.4% (122)	60.9% (98)	
15 years (Group 3)	22.0% (46)	15.5% (25)	
Other (Group 4)	5.7% (12)	4.3% (7)	
Household status			0
Living alone	22.3% (47)	31.5% (51)	
Comorbidity diseases			
Cerebrovascular disease	6.5% (12)	7.6% (10)	58
Cancer	2.7% (5)	3.8% (5)	57
DM	7.0% (13)	10.5% (14)	54
OA	13.8% (26)	15.8% (21)	52
RA	1.1% (2)	0.8% (1)	59
Respiratory disease	4.3% (8)	6.9% (9)	59
Cardiovascular disease	10.8% (20)	15.7% (21)	54
Number of comorbidities	0.5 (0.7)	0.5 (0.8)	64
Employment status			0
Employed	16.1% (34)	6.8% (11)	
Subjective cognitive decline			57
Present	46.4% (84)	49.3% (70)	
Going out at least once a week			0
Yes	95.7% (202)	93.3% (150)	
Frailty status at baseline			0
Robust	64.0% (135)	47.5% (77)	
Pre‐frailty/frailty	36.0% (76)	52.5% (85)	
HL at baseline			0
Mean ± SD	4.2 (0.3)	3.2 (0.5)	
Median (25th and 75th percentiles)	4.0 (4.0, 4.4)	3.2 (3.0, 3.6)	
IADL at baseline (score)			106
Mean ± SD	4.9 (0.5)	4.6 (0.8)	
Median (25th and 75th percentiles)	5.0 (5.0, 5.0)	5.0 (4.0, 5.0)	
Subcategory of IADL			
Can you use public transportation (bus or train) by yourself? (Yes)	94.0% (142)	85.5% (100)	
Can you shop for daily necessities? (Yes)	98.7% (149)	92.4% (110)	
Can you prepare meals by yourself? (Yes)	96.7% (146)	92.4% (110)	
Can you to pay bills? (Yes)	98.7% (149)	95.7% (112)	
Can you handle your own banking? (Yes)	98.0% (148)	95.8% (114)	

DM, diabetes mellitus; HL, health literacy; IADL, instrumental activities of daily living; NMV, number of missing values; OA, osteoarthritis; RA, rheumatoid arthritis.

Data are presented as percentages (numbers), means (standard deviation), and median (25th and 75th percentiles).

At the 1‐year follow‐up, 67.3% and 41.4% of participants in the high and low HL groups, respectively, were classified as robust (*P* < 0.001) (Table S2).

In the logistic regression model predicting pre‐frailty or frailty, higher health literacy remained significantly associated with lower OR of being pre‐frail or frail after 1 year (OR = 0.546, *P* = 0.009), even after adjusting for the confounding factors (Table 2). IADL was also a significant predictor of pre‐frailty or frailty (OR = 0.648, *P* = 0.043) (Table 2).

**Table 2:** Odds ratios of pre‐frailty and frailty at 1 year after baseline according to health literacy at baseline

Variables	Coefficient	Standard error	Adjusted odds ratio*	95% CI		*P*‐value
				Lower	Upper	
Health literacy (0 = low, 1 = high)	−0.623	0.238	0.546	0.336	0.854	0.009
IADL (score)	−0.435	0.215	0.648	0.415	0.975	0.043
Age (year)	0.064	0.019	1.067	1.027	1.108	0.001
Numbers of comorbidities	0.140	0.164	1.150	0.829	1.585	0.395
Years of education, Group 1 [ref]						
Group 2	−0.428	0.329	0.652	0.339	1.234	0.193
Group 3	−0.280	0.407	0.755	0.338	1.674	0.491
Group 4	−0.447	0.638	0.639	0.176	2.193	0.484
Subjective cognitive decline (Yes)	0.617	0.492	1.853	0.716	4.956	0.210
Pre‐frail or frail at baseline (Yes)	1.852	0.227	6.371	4.107	10.010	<0.001
Living alone (Yes)	0.033	0.272	1.033	0.604	1.761	0.904
Employed (Yes)	0.350	0.399	1.419	0.640	3.070	0.380

CI, confidence interval; IADL, instrumental activities of daily living.

Model was adjusted for confounding factors such as age, number of comorbidities, education level, subjective cognitive function, living arrangement (whether participants were living alone), employment status (whether currently employed), and baseline frailty status.

Regression analysis revealed that higher health literacy was positively associated with better IADL outcomes (coefficient = 0.127, *P* = 0.043), even after adjusting for potential confounders including background characteristics (age, comorbidity, cognitive function, and education) and social factors (family status and employment status) (Table S3).

**Figure 2:**
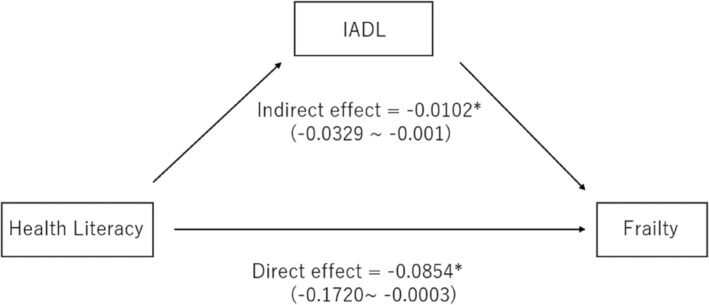
Mediating effects of instrumental activities of daily living (IADL) on the relationship between health literacy and frailty. *P < 0.05.

Causal mediation analysis revealed that IADL mediated 10.7% of the effect of health literacy on frailty status (ACME = −0.010, *P* = 0.030), with both the direct effect (ADE = −0.085, *P* = 0.044) and total effect (estimate = −0.096, *P* = 0.014) remaining significant, even after adjusting for confounders (Table 3, Fig. 2).

**Table 3:** Decomposition of the total effect of health literacy on frailty status at 1 year into direct and indirect effects mediated by instrumental activities of daily living and corresponding 95% CIs

Effect type	Estimates	95% CI		*P*‐value
		Lower	Upper	
ACME (control)	−0.010	−0.032	−0.001	0.030
ACME (treated)	−0.010	−0.031	−0.001	0.030
ADE (control)	−0.086	−0.190	0.000	0.044
ADE (treated)	−0.085	−0.189	0.000	0.044
Total effect	−0.096	−0.198	−0.010	0.014
Prop. mediated (control)	0.109	0.000	0.600	0.049
Prop. mediated (treated)	0.105	0.000	0.590	0.049
ACME (average)	−0.010	−0.032	−0.001	0.030
ADE (average)	−0.085	−0.172	0.000	0.044
Prop. mediated (average)	0.107	0.000	0.590	0.049

ACME, the average causal mediation effect; ADE, the average direct effect.

Model was adjusted for confounding factors such as age, number of comorbidities, education level, subjective cognitive function, living arrangement (whether participants were living alone), employment status (whether currently employed), and baseline frailty status.

## DISCUSSION

Our study confirms that higher health literacy is associated with better IADL outcomes and a reduced risk of progression to pre‐frailty or frailty, as defined by the J‐CHS criteria, even after controlling for various confounding factors including age, comorbidities, cognitive function, and social factors, such as family status and employment.

### Health literacy and frailty

Our results align with those of previous studies, demonstrating the importance of health literacy, including CCHL, in managing health and preventing frailty.^10^, ^11^ The HL scores observed in our study are comparable to those reported in a previous study by Yoshizawa (2021), which documented a mean HL score of approximately four.^10^ This similarity supports the generalisability of our findings to older populations in similar settings. Higher CCHL allows older adults to access, understand, and apply health‐related information more effectively, critical for maintaining physical independence. This is particularly relevant to the instrumental self‐maintenance aspect of IADL with tasks such as using public transportation, shopping for daily necessities, and managing finances. Individuals with higher health literacy may be better able to navigate these complex tasks, facilitating maintenance of autonomy and physical independence, and ultimately reducing frailty risk. Therefore, 67.3% of those in the high HL group remained robust after 1 year, as compared to only 41.4% in the low HL group. Further reinforcing the hypothesis that health literacy plays a key role in mitigating frailty risk, as individuals with better health literacy are more adept at engaging in preventive health behaviours and managing chronic conditions.^9^

### IADL as a mediator

A key finding of this study was the identification of IADL as a significant mediator in the relationship between health literacy and frailty. This IADL subset includes both cognitively and physically demanding essential tasks, such as handling transportation, preparing meals, and managing finances, all of which are critical for maintaining independence in older age.^22^, ^23^ Causal mediation analysis revealed that approximately 10.7% of the effect of health literacy on frailty status was mediated by IADL, indicating that maintaining or improving IADL may be a pathway involved in the effect of health literacy on frailty. The retrospective nature of this study limited its ability to fully account for other health behaviours that may act as mediators. Future prospective studies are needed to evaluate the impact of these additional factors and provide a more comprehensive understanding of the pathways linking health literacy and frailty.

Despite this limitation, our findings suggest that interventions for improving health literacy may indirectly contribute to better IADL performance; thus, preventing or slowing frailty progression. Previous studies have reported an association between IADL decline and higher frailty,^15^ aligning with our finding that IADL was a significant predictor of frailty status after 1 year (OR = 0.648, *P* = 0.043). This highlights the importance of addressing IADL in frailty prevention strategies, particularly among individuals with low health literacy. Interventions that target both health literacy and IADL enhance individuals' abilities to manage their health while maintaining their physical independence.

### Implications for practice

The findings of this study have implications in designing interventions aimed at preventing frailty. First, improving CCHL should be a key component of frailty prevention programs, especially in ageing populations. Health literacy interventions should focus on equipping older adults with the skills needed to access and apply health information to promote healthy behaviours and reduce frailty.

Second, the mediating role of IADL highlights the role of interventions aimed at health literacy improvement and IADL maintenance and enhancement. Programs incorporating physical, cognitive, and social components, aimed at maintaining daily living skills, may be particularly effective in reducing frailty risk. Tailored interventions accounting for unique social and cognitive needs of older adults may be more successful in promoting independence and preventing frailty.

### Limitations

This study has some limitations. First, the observational design limited our ability to establish causality between health literacy, IADL, and frailty. Although mediation analysis provides insights into the potential mechanisms, randomised controlled trials are needed to confirm causal relationships. Moreover, as this study was retrospective, the role of other health behaviours acting as mediators could not be extensively assessed. Future prospective studies are required to address these gaps and to fully evaluate the mediating effects of various health behaviours.

Second, health literacy was assessed using the CCHL Scale, which may not identify all the dimensions of health literacy, especially functional literacy. Moreover, we accounted for various confounding factors including cognitive function and social factors; however, residual confounding may still be present.

Third, the IADL was assessed using the five items from the TMIG‐IC, which may have a ceiling effect. This could potentially limit the ability to detect only small differences in IADL between groups with different health literacy levels. Future studies should consider using additional or alternative measures of IADL providing a wider range of scoring and sensitivity to detect variations in functional independence.

Despite these limitations, this study provides valuable insights into the relationship between health literacy, IADL, and frailty. Future research addressing these limitations will further enhance our understanding of these complex interactions.

### Conclusion

In conclusion, this study demonstrates that health literacy, particularly CCHL, plays a critical role in preventing frailty progression in older adults. Moreover, IADL acts as an important mediator in this relationship, suggesting that interventions aimed at improving health literacy and maintaining daily living skills may be key to reducing frailty risk. As IADL contributed to only a portion of this mediation effect, future research should explore other health behaviours that may serve as mediators. Furthermore, future research should explore how these findings can be translated into practical interventions and policies to promote healthy ageing and prevent frailty in older populations.

## AUTHOR CONTRIBUTIONS

Keisuke Nakamura: project administration, writing – original draft preparation, formal analysis. Tomohiro Sasaki: writing– review and editing. Yoshiharu Yokokawa: investigation. Shinobu Yokouchi: conceptualisation.

## FUNDING INFORMATION

This research received no specific grants from any funding agency in the public, commercial, or not‐for‐profit sectors.

## ETHICS STATEMENT

The study was approved by the ethics committee of Matsumoto City Hospital (protocol number, 03–5; approval date, 22/06/2021).

## PATIENT CONSENT STATEMENT

The need for informed consent was waived due to data anonymisation and the retrospective nature of the study.

## CLINICAL TRIAL REGISTRATION

This study is an observational study and was not registered as a clinical trial as it does not involve any intervention.

## Supporting information


**Table S1.** Patient characteristics at baseline.
**Table S2.** Frailty status after 1 year.
**Table S3.** Linear regression analysis of instrumental activities of daily living (IADL) stratified by health literacy at baseline.

## Data Availability

The data that support the findings of this study are available on request from the corresponding author. The data are not publicly available due to privacy or ethical restrictions.

## References

[1] Makizako H , Nishita Y , Jeong S *et al*. Trends in the prevalence of frailty in Japan: a meta‐analysis from the Ilsa‐j. J Frailty Aging 2021; 10: 211–218. 34105703 10.14283/jfa.2020.68

[2] Murayama H , Kobayashi E , Okamoto S *et al*. National prevalence of frailty in the older Japanese population: findings from a nationally representative survey. Arch Gerontol Geriatr 2020; 91: 104220. 32810734 10.1016/j.archger.2020.104220

[3] Fried LP , Tangen CM , Walston J *et al*. Frailty in older adults: evidence for a phenotype. J Gerontol A Biol Sci Med Sci 2001; 56: M146–M156. 11253156 10.1093/gerona/56.3.m146

[4] Bandeen‐Roche K , Xue Q‐L , Ferrucci L *et al*. Phenotype of frailty: characterization in the women's health and aging studies. J Gerontol A Biol Sci Med Sci 2006; 61: 262–266. 16567375 10.1093/gerona/61.3.262

[5] Xue Q‐L . The frailty syndrome: definition and natural history. Clin Geriatr Med 2011; 27: 1–15. 21093718 10.1016/j.cger.2010.08.009 PMC3028599

[6] Nutbeam D . Health promotion glossary (1998). Health Promot Int 1998; 13: 349–364.

[7] Nutbeam D . Health literacy as a public health goal: a challenge for contemporary health education and communication strategies into the 21st century. Health Promot Int 2000; 15: 259–267.

[8] Vandenbosch J , Van den Broucke S , Vancorenland S , Avalosse H , Verniest R , Callens M . Health literacy and the use of healthcare services in Belgium. J Epidemiol Community Health 2016; 70: 1032–1038. 27116951 10.1136/jech-2015-206910

[9] Lima ACP , Maximiano‐Barreto MA , Martins TCR , Luchesi BM . Factors associated with poor health literacy in older adults: a systematic review. Geriatr Nurs 2024; 55: 242–254. 38070263 10.1016/j.gerinurse.2023.11.016

[10] Yoshizawa Y , Tanaka T , Takahashi K , Fujisaki‐Sueda‐Sakai M , Son B‐K , Iijima K . Impact of health literacy on the progression of frailty after 4 years among community‐dwelling older adults. Int J Environ Res Public Health 2021; 19: 19:394. 10.3390/ijerph19010394. 35010654 PMC8744550

[11] Choi EY , Shin H , Kim S , Lee HY , Kim YS . Limited health literacy increases the risk of frailty among community‐dwelling older adults: longitudinal findings from the Korean frailty and aging cohort study. Geriatr Gerontol Int 2022; 22: 325–331. 35266267 10.1111/ggi.14369

[12] Liu J , Zhu Y , Tan JK , Ismail AH , Ibrahim R , Hassan NH . Factors associated with frailty in older adults in community and nursing home settings: a systematic review with a meta‐analysis. J Clin Med Res 2024; 13: 2382. 10.3390/jcm13082382 PMC11050860 38673654

[13] Wolf MS , Feinglass J , Thompson J , Baker DW . In search of ‘low health literacy’: threshold vs. gradient effect of literacy on health status and mortality. Soc Sci Med 2010; 70: 1335–1341. 20167411 10.1016/j.socscimed.2009.12.013

[14] GJ MD Jr , Mackert M , Becker H . Memory performance, health literacy, and instrumental activities of daily living of community residing older adults. Nurs Res 2012; 61: 70–75. 22166912 10.1097/NNR.0b013e31823b18f4 PMC4839182

[15] Nourhashémi F , Andrieu S , Gillette‐Guyonnet S , Vellas B , Albarède JL , Grandjean H . Instrumental activities of daily living as a potential marker of frailty: a study of 7364 community‐dwelling elderly women (the EPIDOS study). J Gerontol A Biol Sci Med Sci 2001; 56: M448–M453. 11445604 10.1093/gerona/56.7.m448

[16] Ishikawa H , Nomura K , Sato M , Yano E . Developing a measure of communicative and critical health literacy: a pilot study of Japanese office workers. Health Promot Int 2008; 23: 269–274. 18515303 10.1093/heapro/dan017

[17] Fukutomi E , Okumiya K , Wada T *et al*. Importance of cognitive assessment as part of the ‘Kihon checklist’ developed by the Japanese Ministry of Health, labor and welfare for prediction of frailty at a 2‐year follow up. Geriatr Gerontol Int 2013; 13: 654–662. 23170783 10.1111/j.1447-0594.2012.00959.x

[18] Koyano W , Shibata H , Nakazato K , Haga H , Suyama Y . Measurement of competence: reliability and validity of the TMIG index of competence. Arch Gerontol Geriatr 1991; 13: 103–116. 15374421 10.1016/0167-4943(91)90053-s

[19] Satake S , Arai H . The revised Japanese version of the cardiovascular health study criteria (revised J‐CHS criteria). Geriatr Gerontol Int 2020; 20: 992–993. 33003255 10.1111/ggi.14005

[20] Stekhoven DJ , Bühlmann P . MissForest‐‐non‐parametric missing value imputation for mixed‐type data. Bioinformatics 2012; 28: 112–118. 22039212 10.1093/bioinformatics/btr597

[21] Byeon S , Lee W . An introduction to causal mediation analysis with a comparison of 2 R packages. J Prev Med Public Health 2023; 56: 303–311. 37551068 10.3961/jpmph.23.189 PMC10415648

[22] Kawai H , Ejiri M , Imamura K *et al*. Impact of combinations of subscale declines in higher‐level functional capacity on 8‐year all‐cause mortality among community‐dwelling older Japanese adults. Arch Gerontol Geriatr 2023; 114: 105096. 37311368 10.1016/j.archger.2023.105096

[23] Fujiwara Y , Shinkai S , Kumagai S *et al*. Longitudinal changes in higher‐level functional capacity of an older population living in a Japanese urban community. Arch Gerontol Geriatr 2003; 36: 141–153. 12849088 10.1016/s0167-4943(02)00081-x

