# Supplementary material for: Health literacy and frailty: the mediating role of instrumental activities of daily living
Source: Psychogeriatrics. 2025 Feb 10;25(2):e70010. doi: 10.1111/psyg.70010 (PMC11810535; doi:10.1111/psyg.70010)
Supplement: Supplementary file 1 — Table S1. Patient characteristics at baseline. Table S2. Frailty status after 1 year. Table S3. Linear regression analysis of instrumental activities of daily living (IADL) stratified by health literacy at baseline. [file PSYG-25-0-s001.docx]

**Supplemental Materials**

**Table S1. Patient characteristics at baseline**

|  | Followed group | Not Followed group |
| --- | --- | --- |
|  | N=373 | N=372 |
| Age |  |  |
| Median (25th and 75th percentiles) | 78.0 (74.0, 83.0) | 78.0 (74.0, 83.0) |
| Sex  Male  Female | 15.6% (61)  84.4% (312) | 20.1% (75)  79.9% (297) |
| Educational background  9 years (Group 1)  12 years (Group 2)  15 years (Group 3)  Other (Group 4) | 16.0% (60)  59.0% (220)  19.0% (71)  5.0% (19) | 22.9% (86)  56.6% (210)  16.3% (60)  4.2% (16) |
| Household status  Living alone | 26.3% (98) | 21.3% (79) |
| Number of comorbidities |  |  |
| Median (25th and 75th percentiles) | 0.0 (0.0, 1.0) | 0.0 (0.0, 1.0) |
| Employment status  Employed | 12.0% (45) | 11.7% (44) |
| Subjective cognitive decline  Present | 48.7% (154) | 47.3% (176) |
| Going out at least once a week  Yes | 94.4% (352) | 94.6% (352) |
| Frailty status at baseline  Robust  Pre frailty/ Frailty | 56.3% (210)  43.7% (163) | 53.5% (199)  46.5% (173) |

Data are presented as percentages (numbers) and median (25th and 75th percentiles)

**Table S2. Frailty status after 1 year**

|  | High HL group | Low HL group | *P-value* | NMV |
| --- | --- | --- | --- | --- |
|  | N=211 | N=162 |  |  |
| Medical expenses (yen) |  |  | 0.04 | 68 |
| Mean ± SD | 399,320  (674,123) | 460,809  (613,358) |  |  |
| Median (25th and 75th percentiles) | 239,050  (114,350, 421,680) | 295,725  (146,282, 475,202) |  |  |
| Long-term care costs (yen) |  |  | 0.12 | 68 |
| Mean ± SD | 10,449  (68,469) | 40,518  (183,379) |  |  |
| Median (25th and 75th percentiles) | 0 (0, 0) | 0 (0, 0) |  |  |
| Frailty status after one year  Robust  Pre-frail/Frail | 67.3% (142)/  32.7% (69) | 41.4% (67)  58.6% (95) | <0.0001 | 0 |

Abbreviation: NMV, number of missing values; SD, standard deviation

Data are presented as percentages (numbers), means (standard deviation), and median (25^th^ and 75^th^ percentiles).

**Table S3. Linear regression analysis of IADL stratified by health literacy at baseline**

| Variables | Coefficient | Standard  error | 95% CI | | *P*-value |
| --- | --- | --- | --- | --- | --- |
|  |  |  | Lower | Upper |  |
| Health literacy (0=low, 1=high) | 0.127 | 0.062 | 0.003 | 0.250 | 0.043 |
| Age (year) | -0.011 | 0.004 | -0.021 | -0.002 | 0.019 |
| Number of comorbidities | -0.040 | 0.043 | -0.126 | 0.045 | 0.353 |
| Years of education, Group1 [ref] |  |  |  |  |  |
| Group2 | 0.249 | 0.084 | 0.084 | 0.414 | 0.003 |
| Group3 | 0.129 | 0.103 | -0.074 | 0.333 | 0.211 |
| Group4 | -0.048 | 0.161 | -0.365 | 0.269 | 0.765 |
| Subjective cognitive decline (Yes) | -0.070 | 0.118 | -0.302 | 0.162 | 0.556 |
| Pre-frail or Frail at baseline (Yes) | -0.060 | 0.061 | -0.181 | 0.061 | 0.331 |
| Living alone (Yes) | 0.067 | 0.007 | -0.071 | 0.205 | 0.342 |
| Employed (Yes) | -0.012 | 0.096 | -0.200 | 0.176 | 0.901 |

Abbreviation: CI, confidence interval; IADL, Instrumental Activities of Daily Living.

Model was adjusted for confounding factors such as age, number of comorbidities, education level, subjective cognitive function, living arrangement (whether participants were living alone), employment status (whether currently employed), and baseline frailty status.
